# Micropropagation of Lisianthus (*Eustoma grandiflorum* (Raf.) Shinn.) Using Meta-Topolin

**DOI:** 10.3390/biotech15040093

**Published:** 2026-10-13

**Authors:** Marco A. Ramírez-Mosqueda, Alma Armenta-Medina, Obdulia Baltazar-Bernal, José Roberto Bautista-Aguilar, Marco Vinicio Rodríguez-Deméneghi

**Affiliations:** 1 Centro Nacional de Recursos Genéticos, INIFAP, Boulevard de la Biodiversidad No. 400, Rancho las Cruces, 2498, Tepatitlán de Morelos 47600, Jalisco, Mexico; 2 Colegio de Postgraduados Campus Córdoba, Km. 348, Carretera Federal Córdoba-Veracruz, Manuel León, Amatlán de los Reyes 94953, Veracruz, Mexico; 3 Facultad de Ciencias Agrícolas, Universidad Veracruzana, Circuito Gonzalo Aguirre Beltrán, Zona Universitaria, Xalapa 91000, Veracruz, Mexico; 4 Facultad de Ciencias Biológicas y Agropecuarias, Universidad Veracruzana, Peñuela, Amatlán de los Reyes 94945, Veracruz, Mexico

**Keywords:** in vitro propagation, lisianthus, mT, ornamental plant

## Abstract

Lisianthus (*Eustoma grandiflorum*) is an ornamental species of high commercial value due to its diverse flower colors and prolonged vase life. However, lisianthus propagation by seed is limited by its low germination rates and high genetic variability, compromising plant uniformity and production timelines. This study evaluated the effect of meta-Topolin (mT) and of 6-benzylaminopurine (BAP), aromatic cytokinins, on the in vitro micropropagation of lisianthus, aiming to develop an efficient protocol for mass propagation. In vitro established shoots were cultured on Murashige and Skoog (MS) medium supplemented with different concentrations of mT and BAP (0, 0.5, 1.0, or 1.5 mg L^−1^). The parameters assessed were shoot proliferation, shoot length, leaf number, root formation, and root length. Significant differences were observed among treatments. The highest proliferation rate (59.20 shoots per explant) was achieved with 0.5 mg L^−1^ mT, representing a 37-fold increase over the control. With the addition of BAP to the culture medium, the results were inferior. Higher mT concentrations promoted rooting and improved acclimatization success, with the highest survival rate (95%) recorded for plants from the 1.5 mg L^−1^ mT treatment. Low mT concentrations enhanced shoot multiplication but limited root formation, affecting nursery survival. Results demonstrated that meta-Topolin induces a higher shoot number during lisianthus micropropagation, and at higher concentrations, it enables in vitro rooting of the shoots generated during the proliferation stage. This provides a practical alternative for large-scale commercial propagation of this ornamental species.

## 1. Introduction

Ornamental plants are valued for their diverse forms and colors, particularly in their flowers and foliage [1,2]. The demand for flowers and foliage is steadily increasing. *Eustoma grandiflorum* (Raf.) Shinn., commonly known as lisianthus, is a highly valued ornamental species prized for its diverse range of flower pigmentation and notably long postharvest longevity [3,4]. In recent years, the demand for propagules for commercial plantation establishment has risen, especially in countries where this ornamental material has been recently introduced [5,6]. The commercial cultivation of lisianthus in Mexico is a relatively recent practice, and seed-based propagation remains the conventional method employed. However, the exceptionally small seed size—approximately 19,000 seeds per gram—presents considerable challenges in handling and uniform sowing [5,7]. Furthermore, low seed germination rates and high genetic segregation are reported, compromising plant uniformity, flowering time, plant height, and flower number [8,9]. Therefore, innovative propagation techniques are required to generate a greater number of plants in a reduced timeframe.

Plant tissue culture (PTC) is a biotechnological tool permitting the manufacture of thousands of plants within short timeframes and limited spaces [10,11]. In vitro propagation or micropropagation of ornamental species has facilitated the mass production of commercial propagules with high genetic uniformity and pathogen-free status [12,13]. These biotechnological techniques are typically applied to species facing propagation challenges or where conventional methods fail to meet market demand [14,15]. Currently, efforts focus on enhancing plant yields through the use and combination of various plant growth regulators [16,17].

Among the cytokinins widely applied during the shoot multiplication stage, 6-Benzylaminopurine (BAP, also referred to as benzyl adenine or BA) has long been the preferred choice across a broad range of plant species, largely attributed to its proven effectiveness and relatively low cost. Nevertheless, hydroxylated cytokinin analogs, especially those bearing a hydroxyl group at the meta position of the benzyl ring, have progressively gained recognition as functional substitutes [18,19,20]. Of these, meta-Topolin (mT; 6-(3-hydroxybenzylamino) purine) has emerged as the most compelling candidate, with documented positive outcomes on morphogenic responses across multiple plant species [18,21,22]. Numerous positive reports document its use, including enhanced morphogenetic activities, primarily increased shoot proliferation [22,23,24], improved plant vigor due to mitigated physiological disorders [25], and increased survival rates of plants during acclimatization owing to uninhibited root formation [23,24,26]. Additionally, mT has been linked to increased secondary metabolite synthesis [27,28], and recent studies have shown differential gene expression responses compared to BAP [29]. Consequently, the use of mT in plant micropropagation has gained popularity among in vitro propagation experts.

In *E. grandiflorum*, micropropagation has been performed using different types of cytokinins, such as kinetin at 3 mg L^−1^, generating up to 9 shoots after three one-month subcultures [30]. Another study reported the use of 3 mg L^−1^ of 6-benzylaminopurine (BAP) for the formation of 14.9 shoots from nodal segments [31]. When thidiazuron was used in combination with naphthaleneacetic acid (NAA), the formation of 15.2 shoots per explant was achieved [32]. As observed, the number of shoots per explant in these studies is limited and dependent on periodic subculturing, which increases production costs. Therefore, this study aimed to evaluate the effect of mT on the micropropagation of lisianthus (*E. grandiflorum*) for increasing its in vitro multiplication.

## 2. Materials and Methods

### 2.1. Plant Material

In vitro plants of lisianthus (*Eustoma grandiflorum* (Raf.) Shinn. cv. ‘Echo White’) previously established at the Centro Nacional de Recursos Geneticos-INIFAP from apical meristems were used as a source of explants. These in vitro plants were derived from axillary buds in Murashige and Skoog (MS) [33] medium (PhytoTech Labs, Lenexa, KS) supplemented with 1 mg L^−1^ of 6-benzylaminopurine (BAP) (Sigma-Aldrich, St. Louis, MO, USA); upon establishment, plants subjected to five subcultures in MS medium without the addition of any plant growth regulator (PGR), each with a duration of three months, were utilized.

### 2.2. Proliferation of Lisianthus Shoots with Different Cytokinins

Individual shoots of *E. grandiflorum* (1 cm in length) were transferred to MS medium supplemented with 30 g L^−1^ sucrose. In one experiment, different concentrations of meta-Topolin (mT: 0, 0.5, 1.0, and 1.5 mg L^−1^) (Phytotechnology^®^, Lenexa, KS, USA) and different concentrations of BAP, 0, 0.5, 1.0, and 1.5 mg L^−1^ (Sigma-Aldrich, St. Louis, MO, USA), were evaluated. Prior to autoclaving, medium pH was brought to 5.8 using NaOH or HCl, and solidification was achieved by incorporating 2.5 g L^−1^ Phytagel^®^ (Sigma, St. Louis, MO, USA). Sterilization was carried out in an autoclave at 121 °C and 1.5 kg cm^−2^ for 15 min. In total, 35 mL of culture medium was dispensed into glass flasks with a volume of 125 mL. All cultures were incubated at 24 ± 2 °C with a 16-h light/8-h dark cycle, under a photosynthetic photon flux density of 50 µmol m^−2^ s^−1^ delivered by LED lighting. After 45 days of culture, the following traits were evaluated: number of shoots per explant, shoot length, number of leaves formed per explant, and number and length of roots per explant.

### 2.3. Acclimatization Procedure

A total of 40 individual plants per treatment (mT only) with an established root system were rinsed under running water. Rooted plantlets were carefully transferred into 2.5-inch containers (7.2 × 6.8 × 6.8 cm) filled with a sterilized growing substrate consisting of peat moss and agrolite at a 1:3 (*v*/*v*) ratio. Plants were established in a greenhouse fitted with 50% shade cloth, where ambient temperature was maintained at 30 ± 5 °C and relative humidity was kept at 90 ± 5% throughout the acclimatization period. After 60 days of growth in the greenhouse, the survival percentage was evaluated (surviving plants/planted plants × 100).

### 2.4. Statistical Analysis

All experiments followed a completely randomized design with three replicates. A total of 90 explants per treatment were evaluated, distributed in 10 glass jars with 3 explants per jar and three replicates. The data obtained were statistically processed with IBM SPSS Statistics software (version 21). An analysis of variance (ANOVA) followed by Tukey’s multiple comparisons test (*p* ≤ 0.05) was performed to test for statistically significant differences between treatments. Prior to ANOVA, normality and variance homogeneity were checked by Kolmogorov–Smirnov and Levene tests, respectively. When variables did not meet these assumptions, they were transformed to a natural logarithm (ln) to satisfy the requirements for parametric analysis. Results are expressed as mean ± standard error.

## 3. Results

### 3.1. Effect of Meta-Topolin and BAP on In Vitro Micropropagation of Lisianthus

**Figure 1:**
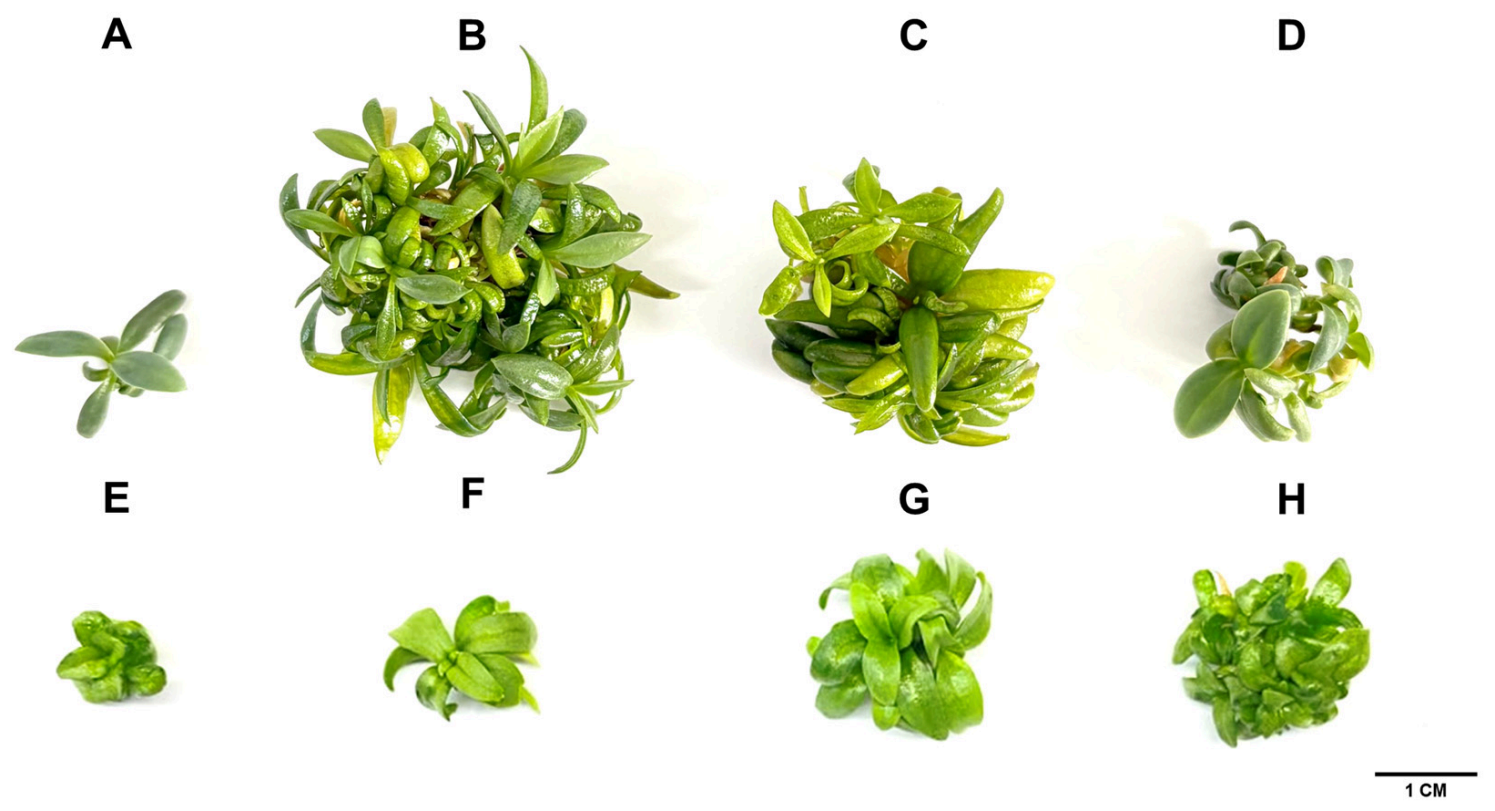
In vitro proliferation of lisianthus (*Eustoma grandiflorum* (Raf.) Shinn.) shoots with different concentrations of meta-Topolin after 45 days of culture. (**A**–**D**) (left to right): 0, 0.5, 1.0, and 1.5 mg L^−1^ mT, respectively; (**E**–**H**) (left to right): 0, 0.5, 1.0, and 1.5 mg L^−1^ BAP, respectively.

**Figure 2:**
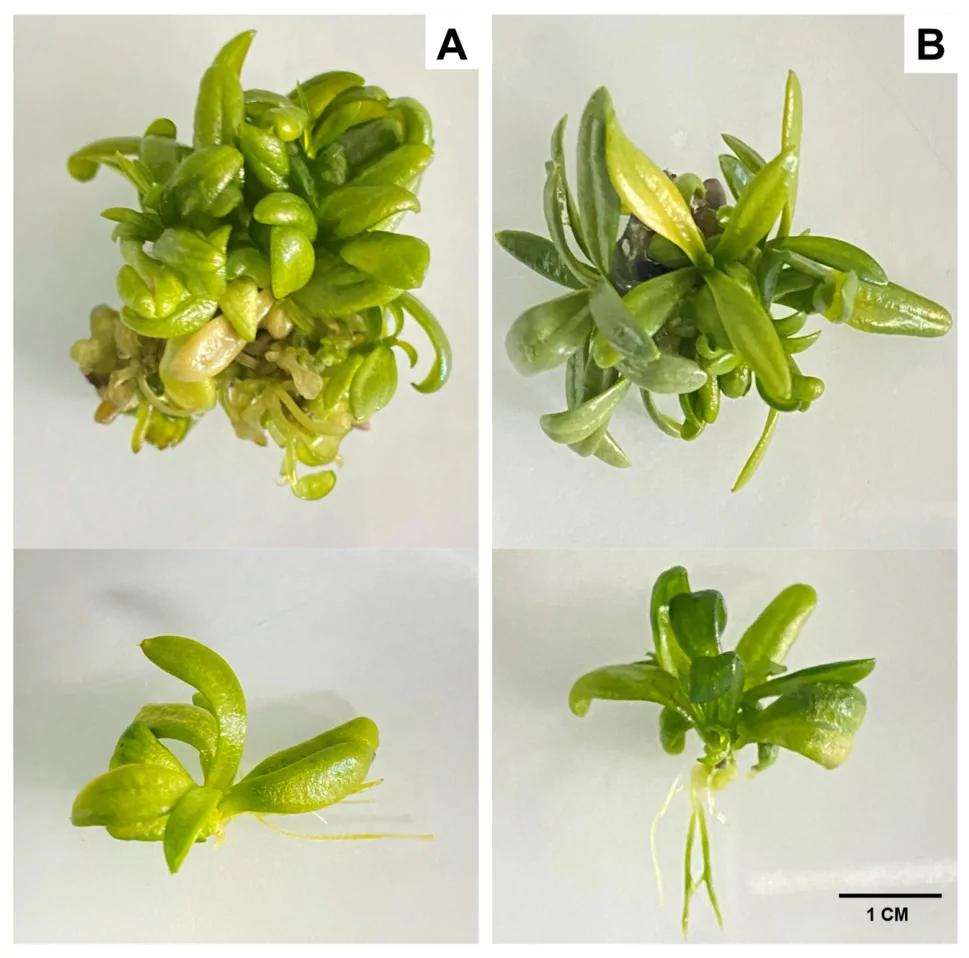
In vitro rooting of lisianthus (*Eustoma grandiflorum* (Raf.) Shinn.) shoots with different concentrations of meta-Topolin after 45 days of culture. (**A**) MS + 0.5 mg L^−1^ of mT; (**B**) MS + 1.5 mg L^−1^ of mT.

After 45 days of culture, significant differences were observed among the evaluated treatments (Table 1). In general, mT generated superior results in the evaluated variables compared to the BAP treatments; shoots showed no visible morphological abnormalities. The highest number of shoots per explant (59.20) was obtained with 0.5 mg L^−1^ mT in the culture medium (Figure 1B), representing a 35- to 40-fold increase compared to the control treatment (1.60 shoots per explant, Figure 1A). The addition of 1.0 and 1.5 mg L^−1^ mT yielded 34.40 and 20.50 shoots per explant, respectively (Figure 1C,D). In contrast, the addition of 1.5 mg L^−1^ BAP generated 21.30 shoots per explant (Figure 1H), 1.0 mg L^−1^ BAP yielded 14.10 shoots (Figure 1G), and 0.5 mg L^−1^ BAP generated 9.35 shoots (Figure 1F). As with mT, the control treatment without BAP produced the lowest number of shoots (Figure 1E). No significant differences were observed in shoot length, as all shoots from the different treatments exhibited similar lengths. In general, increasing mT concentration increased the number of leaves generated (with the exception of the control treatment), whereas increasing BAP concentration resulted in a decrease in this variable. The values were 8.54 leaves per explant with 1.5 mg L^−1^ mT, 6.88 leaves with 0.0 mg L^−1^ mT, 6.31 leaves with 1.0 mg L^−1^ mT, and 5.17 leaves with 0.5 mg L^−1^ mT. For BAP, the following was observed: 8.12 leaves with 0.5 mg L^−1^ BAP, 6.34 leaves with 0.0 mg L^−1^ BAP, 5.94 leaves with 1.0 mg L^−1^ BAP, and 4.32 leaves with 1.5 mg L^−1^ BAP. This indicated that as the number of shoots increased, fewer leaves were produced. The opposite occurred when mT was added to the culture medium. Root formation occurred only in shoots with mT; BAP treatments and controls did not generate roots. The highest number of roots per shoot (1.49) was observed with 1.5 mg L^−1^ mT, followed by 0.60 roots per shoot with 1.0 mg L^−1^ mT, and 0.34 roots per shoot with 0.5 mg L^−1^ mT. Increasing mT concentration increased root number. Root length showed no differences among mT treatments, while the control treatment lacked root formation. Notably, in the treatment with the highest shoot production (0.5 mg L^−1^ mT) only a proportion of shoots developed roots, hindering acclimatization. However, shoots with roots in this treatment were successfully acclimatized (Figure 2A). The treatment with the lowest shoot production (1.5 mg L^−1^ mT) had most shoots with roots, facilitating acclimatization (Figure 2B). This indicated that the addition of BAP inhibits root formation, whereas the use of mT favors root generation and root length even during the in vitro multiplication phase of lisianthus.

### 3.2. Acclimatization

Root number and length drastically influenced plant survival during acclimatization. Plants from the control treatment (without mT) lacked roots and failed to acclimatize. Survival rates were 60.0% (24 plants, 0.5 mg L^−1^ mT), 62.5% (25 plants, 1.0 mg L^−1^ mT), and 95% (38 plants, 1.5 mg L^−1^ mT). Acclimatized plants showed no visible morphological abnormalities. Plants derived from BAP treatments failed to acclimatize due to the absence of a root system.

## 4. Discussion

This study achieved a substantial increase in the number of in vitro lisianthus shoots using meta-Topolin (mT). mT is a cytokinin that has been used in recent years to improve the micropropagation of various ornamental species [20,34]. This plant growth regulator (PGR) has advantages such as generating a higher number of shoots per explant, improving plant physiology, not inhibiting root formation, and increasing secondary metabolite synthesis, compared to cytokinins like 6-benzylaminopurine [35,36]. In this study, 0.5 mg L^−1^ mT increased shoot number per explant 37-fold compared to the treatment without mT (control). However, the large number of shoots obtained hindered proper root system formation in each shoot, affecting the acclimatization process.

The use of mT in the micropropagation of ornamental plants has increased shoot number in species such as: Pink rain lily (*Zephyranthes grandiflora* Lindl.), where 1.5 mg L^−1^ mT generated 4.40 shoots per explant [28]; orchid (*Laeliocattleya canhamiana*), where 1.5 mg L^−1^ mT added to the culture medium yielded 8.27 shoots per explant after two 30-day subcultures [23]; and gladiolus (*Gladiolus hybrida* L.), where 2.0 mg L^−1^ mT generated the highest number of shoots [37]. Additionally, reports confirm the higher efficiency of mT combined with temporary immersion systems in vanilla (*Vanilla planifolia* Jacks.), promoting large-scale micropropagation [24]. Recent studies mention a synergism between mT and BAP that enhances micropropagation in ornamental plants: for example, Shaheen et al. [38] obtained a maximum of 17 shoots per explant in Gerbera (*Gerbera jamesonii* Bolus ex Hook. F.) by combining (mT and BAP, 2.0 mg L^−1^ each). In goldenberry (*Physalis peruviana* L.), Vargas et al. [39] observed a synergism between 0.5 mg L^−1^ mT and 2.0 mg L^−1^ BAP, promoting the highest shoot regeneration rate (62.96%) from cotyledons. In this study, a maximum of 59.20 shoots per explant was achieved with 0.5 mg L^−1^ mT, indicating a high shoot production without combining mT with another cytokinin or auxin, potentially reducing operational costs.

On the other hand, the use of mT in plant micropropagation does not inhibit root formation in generated shoots, unlike other cytokinins like BAP [40,41]. Several independent studies have documented spontaneous rhizogenesis during the shoot multiplication stage when mT was employed, even in the absence of exogenous auxins [23,41], an outcome rarely observed with conventional cytokinins such as BAP. In this study, root formation occurred in the same lisianthus (*E. grandiflorum*) shoot multiplication stage, allowing high survival rates during acclimatization. However, this was observed only in mT concentrations that didn’t produce a large number of shoots per explant. In 0.5 mg L^−1^ mT, generating 59.20 shoots per explant, not all shoots rooted, hindering acclimatization. To maximize the high multiplication rate observed in this study with 0.5 mg L^−1^ mT, a prior rooting phase is required to ensure maximum plant acclimatization. In vitro generation of a limited number of shoots favors root formation due to nutrient availability and the absence of inter-shoot competition. The ultimate benchmark of any micropropagation protocol lies in the ability of regenerated plantlets to survive and establish themselves under greenhouse conditions, making ex vitro acclimatization a critical determinant of commercial viability [42,43]. This study’s results can contribute to increased lisianthus (*E. grandiflorum*) propagule production for commercial plantations.

## 5. Conclusions

The use of meta-Topolin (mT), an aromatic hydroxylated cytokinin, significantly increased the number of shoots per explant obtained in micropropagation of lisianthus (*Eustoma grandiflorum* (Raf.) Shinn.). The concentration of 0.5 mg L^−1^ mT generated an average of 57.6 more shoots per explant compared to the treatment without mT. However, this concentration showed a drastic decrease in root number per shoot, hindering acclimatization. To capitalize on the high multiplication rate observed with 0.5 mg·L^−1^ mT in this study, a prior rooting phase is necessary to ensure optimal plantlet acclimatization. Higher mT concentrations favored root number and length but decreased shoot production. These preliminary results suggest the potential for large-scale micropropagation of this valuable ornamental crop to obtain commercial propagules; however, further evaluation of repeated multiplication cycles, production costs, genetic fidelity, and post-acclimatization performance is required.

## Figures and Tables

**Table 1:** Effect of meta-Topolin on the micropropagation of lisianthus (*Eustoma grandiflorum* (Raf.) Shinn.) after 45 days of culture.

PGR (mg L^−1^)	Shoot Number/Explant	Shoot Length (cm)/Explant	Number Leaves/Explant	Number Roots/Explant	Root Length (cm)/Explant
meta-Topolin (mT)					
0.0	1.60 ± 0.22 f	0.81 ± 0.07 a	6.88 ± 0.36 b	0.0 ± 0.00 d	0.00 ± 0.00 b
0.5	59.20 ± 2.41 a	0.78 ± 0.08 a	5.17 ± 0.29 b	0.34 ± 0.03 c	0.42 ± 0.05 a
1.0	34.40 ± 3.15 b	0.82 ± 0.07 a	6.31 ± 0.83 ab	0.60 ± 0.07 b	0.41 ± 0.05 a
1.5	20.50 ± 1.91 c	0.75 ± 0.08 a	8.54 ± 0.24 a	1.49 ± 0.12 a	0.41 ± 0.05 a
6-benzylaminopurine (BAP)					
0.0	1.39 ± 0.14 f	0.83 ± 0.08 a	6.34 ± 0.32 b	0.0 ± 0.00 d	0.00 ± 0.00 b
0.5	9.35 ± 1.46 e	0.74 ± 0.07 a	8.12 ± 0.19 a	0.0 ± 0.00 d	0.00 ± 0.00 b
1.0	14.10 ± 1.17 d	0.86 ± 0.06 a	5.94 ± 0.72 b	0.0 ± 0.00 d	0.00 ± 0.00 b
1.5	21.30 ± 1.22 c	0.78 ± 0.05 a	4.32 ± 0.28 a	0.0 ± 0.00 d	0.00 ± 0.00 b

Numbers represent the mean ± SE (standard error). Means with different letters are significantly different (Tukey, ρ ≤ 0.05).

## Data Availability

The original contributions presented in this study are included in the article. Further inquiries can be directed to the corresponding author.

## Abbreviations

The following abbreviations are used in this manuscript:

mT	meta-Topolin
BAP	6-Benzylaminopurine
MS	Murashige and Skoog
PTC	Plant tissue culture
NaOH	Sodium hydroxide
HCl	Hydrochloric acid
PGR	Plant growth regulator
